# The burden of headache disorders in India: methodology and questionnaire validation for a community-based survey in Karnataka State

**DOI:** 10.1007/s10194-012-0474-1

**Published:** 2012-08-22

**Authors:** Girish N. Rao, Girish B. Kulkarni, Gopalkrishna Gururaj, Kavita Rajesh, D. Kumaraswamy Subbakrishna, Timothy J. Steiner, Lars J. Stovner

**Affiliations:** 1 Department of Epidemiology, National Institute of Mental Health and NeuroSciences, Bangalore, 560-029 India; 2 Department of Neurology, National Institute of Mental Health and NeuroSciences, Bangalore, 560-029 India; 3 Department of Bio-Statistics, National Institute of Mental Health and NeuroSciences, Bangalore, 560-029 India; 4 Department of Neuroscience, Imperial College London, London, UK; 5 Department of Neuroscience, Norwegian University of Science and Technology, Trondheim, Norway; 6 Norwegian National Headache Centre, St. Olavs University Hospital, Trondheim, Norway

**Keywords:** Primary headache disorders, Migraine, Tension-type headache, Epidemiology, Burden of disease, Methodology, Survey, Validation, Global Campaign against Headache, India

## Abstract

Primary headache disorders are a major public-health problem globally and, possibly more so, in low- and middle-income countries. No methodologically sound studies of prevalence and burden of headache in the adult Indian population have been published previously. The present study was a door-to-door cold-calling survey in urban and rural areas in and around Bangalore, Karnataka State. From 2,714 households contacted, 2,514 biologically unrelated individuals were eligible for the survey and 2,329 (92.9 %) participated (1,103 [48 %] rural; 1,226 [52 %] urban; 1,141 [49 %] male; 1,188 [51 %] female; mean age 38.0 years). The focus was on primary headache (migraine and tension-type headache [TTH]) and medication-overuse headache. A structured questionnaire administered by trained lay interviewers was the instrument both for diagnosis (algorithmically determined from responses) and burden estimation. The screening question enquired into headache in the last year. The validation study compared questionnaire-based diagnoses with those obtained soon after through personal interview by a neurologist in a random sub-sample of participants (*n* = 381; 16 %). It showed high values (>80 %) for sensitivity, specificity and predictive values for any headache, and for specificity and negative predictive value for migraine and TTH. Kappa values for diagnostic agreement were good for any headache (0.69 [95 % CI 0.61–0.76]), moderate (0.46 [0.35–0.56]) for migraine and fair (0.39 [0.29–0.49]) for TTH.The survey methodology, including identification of and access to participants, proved feasible. The questionnaire proved effective in the survey population. The study will give reliable estimates of the prevalence and burden of headache, and of migraine and TTH specifically, in urban and rural Karnataka.

## Introduction

Sound and reliable epidemiological information is the essential basis of health-care needs assessment, of planning and organizing health-care services and of resource allocation [1].

In a world where resources are limited, headache disorders are unrecognised as a public-health priority and fare badly in the queue for resource allocation, despite clear evidence of the ill-health, disability and economic burdens they impose [2, 3]. There is evidence that this is no less so in low- and middle-income (LAMI) countries [2], while being exacerbated by the general lack of resources. However, reliable population-based data, sufficient to support the argument for change, are limited or not available in these countries [4]. Much information from other parts of the world is difficult to interpret, and comparisons or extrapolations are limited by methodological variations in study design, in sampling (method, representativeness and sample size), in accessing and engaging participants (personal interview, telephone or postal survey), in phrasing the screening and diagnostic questions, in timeframe of headache (life-time, 1 year, 3 months, etc.) and in the specific diagnoses considered [5, 6]. Consequently, reported prevalence rates show substantial variation [6]. Further, most such information comes from developed countries [2, 4, 6].

From an Indian perspective, few studies describe the epidemiology of headache disorders. Previously, these disorders have been investigated only within larger neuroepidemiological surveys that have neither focused on headache nor used internationally accepted criteria for headache diagnoses [7]. This is the background for the present population-based study in the south Indian state of Karnataka, performed with support from the nongovernmental organization *Lifting The Burden* as a project within the Global Campaign against Headache [8]. The study was designed to overcome the limitations of previous Indian studies, and with the principal objective of estimating the prevalence and burden of the headache disorders of public-health importance (migraine, tension-type headache [TTH] and medication-overuse headache [MOH]) in a representative sample of the adult general population. The methodology, procedures and steps of the study, and the results of the diagnostic questionnaire validation, are presented in this report.

## Methodology

### Ethics

The Institutional Ethics Committee of National Institute of Mental Health and Neurosciences (NIMHANS), Bangalore, India approved the study protocol. Informed consent was obtained from participants before interviews commenced. In addition, to obtain wide acceptance of the study, a brief overview of the study and intended interviews, and their purpose and objectives, were presented to local community leaders, and their queries answered and doubts clarified.

### Population of interest

The adult population between 18 and 65 years in both urban and rural Bangalore was the population identified for the study. Institutional households (paying-guest accommodation, hostels, etc.) and immigrants (defined as staying for <6 months in the household and locality) were excluded, as were bachelor households (those containing two or more bachelors belonging to different families staying together, whether or not sharing the preparation or partaking of food) on the basis that they were not permanent residents. Those not conversant with the local language (Kannada) were also excluded.

### Study design

The study was a cross-sectional survey, using cluster sampling among urban and rural populations representative of the population of Karnataka State.

### Sample size

Applying an expected prevalence of headache of 35 %, based on review of previous studies [6], and with 95 % confidence and 10 % relative error margins, the minimum sample requirement was 1,000 biologically unrelated individuals from each (urban and rural) stratum.

### Study area

The study was undertaken in both urban and rural areas of Bangalore. For the urban component, after excluding predominantly commercial areas within Bangalore city, specific geographical areas with relatively stable population consisting of a mix of upper, middle and lower classes were listed and one area (Kemepegowdanagara [http://g.co/maps/ungmu]) was randomly selected. For the rural component, one revenue subdivision (taluka) in Bangalore rural district was randomly chosen and, from a list of villages therein obtained from Census 2001 data, two villages (Uyamballi and Doddaaladahalli [http://g.co/maps/9gbnt]), located almost 110 km from Bangalore city and with 500 and 800 households respectively, were randomly selected.

### Sampling

**Fig. 1:**
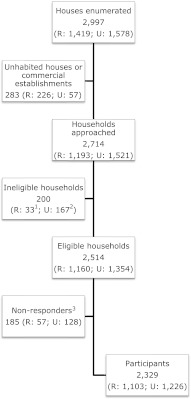
Study population and participation (*U* urban, *R* rural). *1* Ineligible as these households had no persons within the age range 18–65 years). *2* Households only with persons <18 or >65 years (*n* = 19), not knowing local language (*n* = 62) or who are bachelors staying together (*n* = 86). *3* Includes both refusals (nil rural, 25 urban) and non-participants (interview not possible even after three appointments, at least two of them being on Sundays or holidays)

An individual household (defined as a group of people living together and sharing a kitchen) was considered as the Primary Sampling Unit. Local residential maps were developed to identify area boundaries and household distribution, and all households then given unique numbers. Uninhabited or abandoned houses, institutional households and commercial establishments were excluded for survey purposes (see Fig. 1).

In each surveyed locality, the first household was identified using the random direction method. The survey team, on reaching the centre of the locality, numerically marked the major roads along all four directions. One road was selected corresponding to the last digit of a currency note. Households in this road were visited consecutively (door-to-door) by knocking at their doors unannounced (cold-calling). The first household on the right side of the road became the first in the survey. The second survey household was the one on the left side of the first, and the third was the household on the left side of the second. This process continued until the requisite sample was achieved.

All households approached were accounted for in the survey (Fig. 1). When the doors were locked, or no responsible person (either the head of the household, or any other adult member of the household who could provide reliable information for all members) was available, the household was visited again on the same evening or within the next 3 days (‘mop-up visits’). When necessary, the co-operation of neighbour households was sought to make contact with the required household members. Extra efforts were made to include such households during the last week of the survey in each area (‘final mop-up round’).

In each surveyed household, a responsible person was asked to list all members (defined as residing there for >6 months) in a specific order (oldest male first, followed by oldest female, and so on to the youngest male and youngest female). From this list, one individual aged between 18 and 65 years, male or female, was randomly selected as the participant in the study. Those who were not conversant in the local language (Kannada) were excluded and replaced by further random selection from the household list. The selected participant was interviewed immediately if present and consenting; when he or she was not present, an appointment was made for a return visit at a convenient time. In case of refusal, only age and socio-economic status were documented along with the key reason(s) for refusal. No replacements were made at the household level for selected respondents who withheld consent or failed to keep three appointments, two being on Sundays or holidays; these people were listed as non-participants (Fig. 1).

### The study instrument

The survey instrument was a structured questionnaire developed for the study after a review of the literature; it has meanwhile been used, in appropriate translations, in community-based Global Campaign surveys of headache disorders in China [9] and Russia [10].

The first part contained socio-demographic questions (on age, gender, education, income). The Kuppuswamy scale for classification of socio-economic status of individuals [11] was utilized after updating income levels to the year 2008. The second part began with the screening questions for life-time (“Have you ever had a headache?”) and 1-year prevalence of headache (“Have you had headache during the last year?”), and these were asked of all participants; it concluded, for those answering “yes” to the second question, with an enquiry into frequency of headache. Participants reporting headache on ≥15 days/month were asked a subset of questions designed to diagnose MOH. The third part consisted of diagnostic questions for migraine and TTH based on the International Classification of Headache Disorders, 2nd edition (ICHD-2) [7]. Before these were applied, an introductory question asked whether all headaches experienced by the participant were of one or >1 type: those with >1 type were asked to keep in mind only the subjectively most bothersome type. The fourth part comprised questions on headache with a 1-day timeframe (“Did you have headache yesterday?”), and duration, intensity and burden of that headache. The fifth part enquired into health-care utilization for headache. The sixth was a single question on willingness to pay (WTP) for effective headache treatment (“I want you to imagine that there is a treatment you can buy. If you take it, your headaches will not bother you. I am going to ask you how much you would be willing to pay for this treatment”). The seventh part assessed disability burden using the Headache-Attributed Lost Time (HALT) index [12]. The eighth was the World Health Organization’s 8-item Quality-of-Life scale of (WHOQoL-8) [13], and was applied to all participants to permit comparison between those with and without headache.

All parts were translated into Kannada following *Lifting The Burden*’s translation protocol [14].

### Diagnostic algorithm

Diagnoses were not made by the interviewers; instead, responses to the diagnostic question set were fed into an algorithm developed for the purposes of the study by LJS and TJS. This algorithm was converted to a computer program in Epi-info version 3.5 [15].

In participants reporting more than one headache type during the last year, only the subjectively most bothersome was diagnosed. As for the headache yesterday, it was asked only whether this had been of the same type as the most bothersome headache. Cases were first identified of headache occurring on ≥15 days/month and, amongst them, those with medication overuse (MOH). The algorithm was applied to the remainder of the participants, applying ICHD-2 criteria [7] in hierarchical sequence first for migraine, then for TTH, then for probable migraine and finally for probable TTH. Cases remaining were considered unclassifiable. In the later analysis, migraine and probable migraine were grouped as all-migraine, and TTH and probable TTH as all-TTH.

### Selection and training of interviewers

Six field interviewers with a background in social sciences, and experienced in the methods and techniques of community-based surveys, were recruited for data collection. They were trained through multiple methods: lectures, demonstrations, supervised and independent skill-development sessions. The *Manual for descriptive studies* developed by NIMHANS [16] for epidemiological studies in developing countries was a key resource document. Training was conducted in the local language (Kannada). Interviewers were taken through the final version of the questionnaire so that they understood the nature and sequence of questions, and which were of particular importance (e.g., the screening and diagnostic questions). As hands-on instruction, they undertook mock and supervised interviews, and, finally, independent interviews on patients with and without headache in the outpatient department of NIMHANS. These steps aimed to standardize the method of data collection, reducing intra- and inter-observer variability.

### Pre-pilot and pilot studies

The pre-pilot study was undertaken in the clinical setting. A neurologist and headache expert (GBK) administered the study questionnaire to 40 purposively selected individuals to demonstrate acceptability and inoffensiveness of the questionnaire.

The pilot study was population based, applying the full study methodology for selection of participants and data collection. It was conducted in both rural and urban areas, and interviewed 224 individuals aged 18–65 years, representative of the different socioeconomic strata. Its objectives were to test procedures for selection of households and of participants within households, field test the study instrument, estimate the participation rate and identify and solve logistic problems that might occur in the main study.

During the pilot study, the KISH method [17] of selecting participants from individual households gave a very low interview-completion rate (34 %) compared with simple random selection from the household member list (89 %). Hence, it was decided to use the latter in the main study. Experience from the pilot study also led to editing of the questions on headache characteristics and willingness to pay to suit the cultural context. HALT index and WHOQoL questions needed more explanations to the interviewee to clarify the concepts behind them and their foci of enquiry.

### Main study

The trained interviewers, calling door-to-door in the manner described above, visited households in the identified urban and rural communities. They conducted interviews during morning hours and at mutually convenient times by appointment. In the rural area, the field staff took up temporary residence nearer to the study locality to ensure that early morning, late evening, Sunday and holiday appointments were not missed.

### Quality assurance

Weekly monitoring meetings were held at NIMHANS, Department of Epidemiology to review the progress of work. The investigators conducted monthly refresher training for the field staff. Surprise field visits were undertaken, and a 10 % sub-sample of participants were re-interviewed within 3 weeks of first interview. The discrepancies were very few and, as they pertained to the nature of costs incurred, they were not considered to be significant.

### Validation study

Diagnostic validation required that a sub-sample of participants were re-interviewed, soon after, by a headache expert (GBK) who was blind to the questionnaire diagnoses and applied his clinical skills as well as ICHD-2 criteria to make his own “gold-standard” diagnoses. The 15 % sub-sample was randomly selected. Reviews were undertaken within 3–6 weeks of the primary interviews.

In the beginning, letters were sent inviting selected participants from urban areas to NIMHANS and those from rural areas to its extension services. Because of very poor response, general health camps were held in both urban and rural areas, in the surveyed localities (with a maximum walking distance of 1 km). They were conducted on holidays or Sundays by arrangement with local leaders. The response among selected participants was only marginally better (fewer than one quarter attended, the majority of whom were badly affected by migraine). However, turnout was huge as people found it a good opportunity to seek care for general medical problems and/or other neurological problems. This posed logistic difficulties in planning the camps and, especially, to arrange supplies of free medication.

Finally, the door-knock approach was adopted, with the headache expert calling a second time at the houses of selected participants, and this provided acceptable coverage rates.

### Data entry

Completed survey forms, as received, were scrutinized for accuracy, completeness, inconsistencies, wrong or illegible markings and missed entries first by the team coordinator (Mr. Lokesh) and then by study team member (KR). When discrepancies were observed, study team members (primarily KR but also GNR and GG) discussed them with the field research officers during the weekly meetings. Minor discrepancies (wrong markings, illegible entries) were corrected after discussion but, if there were more major discrepancies, or important data were not collected, the team coordinator was asked to re-visit the household to ascertain what was correct. In fact there were no major discrepancies from the second week of the survey until completion.

Data were entered into a secure database using Epiinfo [15]. A random 10 % of the entered data was cross checked against the original forms. There were very few wrong entries, and therefore no concerns about the overall accuracy of data entry. The high level of accuracy was possible due to the ‘check-conditions’ and ‘jump-conditions’ included in developing the data entry form in Epiinfo.

### Statistical analysis

Analyses were undertaken using Epiinfo [15] and SPSS [18]. In the validation study, sensitivities, specificities and positive (PPV) and negative predictive values (NPV) [19] and Cohen’s Kappa scores [20] were calculated using freely available on-line statistical calculators.

## Results

A total of 2,714 households (1,521 urban 1,193 rural) were visited, in which 12,253 individuals were enumerated. Of 2,514 biologically unrelated individuals (1,354 [53.9 %] urban; 1,160 [46.1 %] rural) who were eligible and selected for interview, 2,329 consented to participate (participation rate 92.6 %) (Fig. 1). Of the 185 non-participants, only 25 (all urban) refused to be interviewed; the main reason for non-participation was missing three agreed appointments to complete the survey interview. A majority of non-participants were male (>75 %) and from urban area (69 %). Of participants, 49 % were male, with little difference between urban and rural areas (Table 1).

**Table 1:** Comparison between responders and non-responders

	Responders			Non-responders^a^		
	Urban	Rural	Total	Urban	Rural	Total
*n*	1,226	1,103	2,329	128	57	185
Male gender (%)	49.8	48.1	49.0	75	77.2	75.7
Age (mean ± SD)	36.7 ± 12.4	39.5 ± 13.0	38.0 ± 12.8	35.4 ± 11.1	39.0 ± 12.1	37.0 ± 11.8
Socio-economic status (%)						
Upper (I)	2.0	0.0	1.1	3.0	0.0	2.2
Upper middle (II)	27.7	4.2	16.6	30.2	6.5	23.2
Lower middle (III)	44.0	15.7	30.6	42.2	17.6	34.6
Upper lower (IV)	25.4	77.2	49.9	23.1	72.1	38.4
Lower (V)	0.8	2.9	1.8	1.5	3.8	2.2

^a^Includes both refusals (*n* = 25, all urban) and non-participants (interview not possible even after three appointments, at least two of them on Sundays or holidays)

**Fig. 2:**
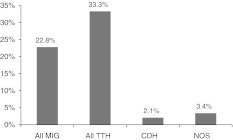
Prevalence of primary headache disorders according to expert diagnosis in the validation sample (*n* = 381) (*All-MIG* migraine + probable migraine, *All TTH* tension-type headache + probable tension-type headache, *CDH* headache occurring on ≥15 days/month, *NOS* not otherwise specified [unclassifiable])

The average time needed for interviews was approximately 40 min (range 20–65 min).

In the validation sub-sample were 482 selected participants of whom 381 (79 % [76.5 % urban; 82.0 % rural] of those selected and 16 % [16 % urban; 17 % rural] of all participants) were re-interviewed. The other 101 could not participate because either they were not available or they refused.

Sensitivity, specificity, PPV and NPV for any headache, and specificities and NPVs for migraine and TTH, were ≥79 %, whereas the sensitivities and PPVs for migraine, TTH and headache on ≥15 days/month were lower (55–63 %) (Table 2). Kappa values showed good agreement (0.69; 95 % CI 0.61–0.76) between headache-expert and algorithmic diagnoses for any headache, moderate agreement (0.46; 0.35–0.56) for migraine and fair agreement (0.39; 0.29–0.49) for TTH. There were only minor variations between urban and rural areas.

**Table 2:** Estimates of questionnaire diagnostic accuracy for different headache types

	Any headache	Migraine	TTH	CDH
Sensitivity, % (95 % CI)	88 (83–91)	63 (52–72)	57 (48–65)	57 (48–65)
Specificity, % (95 % CI)	81 (74–87)	85 (81–89)	81 (76–86)	82 (76–86)
Positive predictive value, % (95 % CI)	89 (84–92)	55 (45–65)	61 (52–69	61 (52–69)
Negative predictive value, % (95 % CI)	80 (73–86)	89 (85–92)	79 (74–84)	79 (74–85)
Kappa value (95 % CI)	0.69 (0.61–0.76)	0.46 (0.35–0.56)	0.39 (0.29–0.49)	0.51 (0.24–0.79)

Migraine and TTH include probable migraine and probable TTH, respectively

In the validation study, the prevalence of migraine was 22.8 %, the prevalence of TTH was 33.3 % and the prevalence of headache occurring on ≥15 days/month was 2.1 %. There were unclassifiable headaches in 3.4 % (Fig. 2) . The proportions were higher in rural areas (Migraine: 27.5 % in Rural and 18.6 % in Urban; TTH: 34.1 % in Rural and 32.7 % in Urban; CDH: 3.3 % in Rural and 1.0 % in Urban).

Results from the main study will be presented in future publications.

## Discussion

This study is the first population-based survey in India to estimate the prevalence of primary headache disorders, and the first to measure headache-attributed burden. To overcome the several methodological limitations of earlier studies, we used essential principles of scientific survey methodology, ensured geographical and socioeconomic representativeness, adopted probability methods of random sampling, used carefully trained interviewers, had a high participation rate (>90 %), included the major primary headache disorders (migraine and TTH) and MOH, employed ICHD-2 diagnostic criteria [7], validated the diagnostic questionnaire against headache-expert diagnoses, estimated life-time, 1-year and 1-day prevalences and quantified headache-attributed burden in a number of ways. We also imposed very stringent quality control.

Most previous studies from India have been clinic-based, but some neuroepidemiological surveys included a question on the presence or absence of headache and/or migraine. The largest, the Bangalore Urban Rural Neuroepidemiological (BURN) study [21], adopted the two-stage method of screening and subsequent confirmation by neurologist [16, 22] and, despite the unfeasibly low prevalence estimates (headache 1.1 % and migraine <1 %), headache was the most commonly reported neurological problem. The findings of the present study will be reported elsewhere but, meanwhile, the validation study, in a small but random sub-sample of the participants of the main study, indicates that headache is much more prevalent in India, and in accord with findings from other parts of the world [6].

Researchers in developing countries such as India face challenges in undertaking community-based studies: not only are there resource constraints, but the country is large and the population highly diverse in culture, education and wealth. While technological limitations restrict the use of telephone interviews or web-based surveys, door-to-door visiting remains the only effective way of selecting and achieving access to a representative sample of the population. This method consumes resources that are not readily available. At the same time, there are few headache specialists or neurologists available, and even fewer who are willing to participate in population surveys. The approach we adopted—of survey by trained lay interviewers with diagnostic validation by a specialist in a sub-sample of those surveyed—is a practical option. Use of a questionnaire that has been employed and validated in surveys in different countries, and of a diagnostic algorithm based on ICHD-2 criteria, ensures comparability between studies. The questionnaire developed for this survey was meanwhile used in similar population-based studies in China [9] and Russia [10].

Diagnosis is of crucial importance, central to all epidemiological surveys. The clinimetric properties of the questionnaire gave acceptable levels of agreement for any headache disorder and for migraine in these studies [9, 10], as it did here (Tables 2 and 3). In all cases, sensitivity was lower than specificity, which is also a characteristic of ICHD-2 [7]. High specificities ensure that most (>80 %) diagnosed cases were correct, while lower sensitivities, especially for TTH, may mean that under-diagnosis occurred. With TTH this is particularly true because, on the one hand, ICHD-2 diagnostic criteria for TTH are especially inclined towards insensitivity and, on the other, many cases of TTH are mild and/or infrequent and therefore not readily reported. From a public-health perspective, however, this may not greatly matter: such cases are not associated with significant health-care need.

**Table 3:** Properties of the diagnostic instrument in this and other studies

Study	Migraine		TTH	
	Sensitivity (%)	Specificity (%)	Sensitivity (%)	Specificity (%)
Yu et al. [9]	83	99	51	99
Ayzenberg et al. [10]	77	82	64	91
Present study	63	85	57	81

It is a particular challenge in population surveys to obtain reliable and consistent answers to questions related to duration of headache attacks, quality and severity of pain, influence of physical activity, photophobia, phonophobia and other associated symptoms. Yet diagnosis hinges on these answers. Well-trained and interested interviewers are essential, who understand which questions are of special importance and why, and we strongly recommend involving them from the conceptual stages of the survey. Not all headache types can be identified by standard survey methods—and not all may be important to the survey objectives. These require clear definition: for example, a survey of headaches of public-health importance needs to identify only migraine, TTH and headache on ≥15 days/month (including MOH). (This, we believe, is generally true: it may be that chronic post-traumatic headache and chronic post-meningitic headache should be considered in some countries). Multiple headache types in the same individual cause difficulties for respondents and interviewers unless these are clearly separated. It may be too much to expect detailed questions to be answered accurately, or at all, on more than one headache type, and surveys should not attempt to be overambitious in this respect. The “most bothersome headache” approach addresses this, is parsimonious and has also been successfully adopted in other studies [9, 10, 23]. Recall bias is an important and potentially problematic factor, and inclusion of questions on headache yesterday addresses this directly. It produces a parallel dataset that should be consistent with the main dataset; if it is not, recall bias must be suspected. But more than this, the 1-day timeframe—provided that the sample size is large enough—gives a very clear view of population burden.

In all of this, how important is quality assurance? There must be some quality assurance measures taken, but it is difficult to know what level of investment in these is maximally efficient. We chose to put a substantial part of the available resources into them—weekly monitoring meetings, monthly refresher field-staff training, surprise field visits, re-interview of a 10 % sub-sample of the participants and rigorous data-entry checks. We believe that these constituted a major strength of the study, and that the results, when reported, will be robust because of them. The rate of detected errors was low, but quality assurance measures do not merely detect errors—they also prevent them.

The main weakness of the study is that it was performed in only one Indian State. In a large and culturally diverse country, caution must be exercised in generalizing the results to other States and in extrapolating to the population of the country. The study focused on primary headache disorders, and did not include secondary headaches other than MOH. It did not enquire about hypertension (often unrecognized and untreated in India) or past history of head injury (common in India). These two conditions could have contributed to the observed prevalence of headache, and future epidemiological surveys could specifically enquire and incorporate them into the validity phase of the study.

In conclusion, this study is the first community-based scientific survey of primary headache disorders in a large urban/rural population of India in which diagnoses are based on ICHD-2 criteria. Several methodological limitations of earlier studies were identified and overcome. The results will follow in later publications.
